# *Acinetobacter baumannii*: Epidemiological and Beta-Lactamase Data From Two Tertiary Academic Hospitals in Tshwane, South Africa

**DOI:** 10.3389/fmicb.2018.01280

**Published:** 2018-06-12

**Authors:** Michelle Lowe, Marthie M. Ehlers, Farzana Ismail, Gisele Peirano, Piet J. Becker, Johann D. D. Pitout, Marleen M. Kock

**Affiliations:** ^1^Department of Medical Microbiology, Faculty of Health Sciences, University of Pretoria, Pretoria, South Africa; ^2^Department of Medical Microbiology, Tshwane Academic Division, National Health Laboratory Service, Pretoria, South Africa; ^3^Departments of Microbiology, Immunology, Infectious Diseases and Pathology and Laboratory Medicine, Cumming School of Medicine, University of Calgary, Calgary, AB, Canada; ^4^Division of Microbiology, Calgary Laboratory Services, Calgary, AB, Canada; ^5^Research Office, Faculty of Health Sciences, University of Pretoria, Pretoria, South Africa

**Keywords:** *Acinetobacter baumannii*, MDR, South Africa, PFGE, MLST, *bla*_OXA-23-like_

## Abstract

*Acinetobacter baumannii* is an opportunistic pathogen that is increasingly responsible for hospital-acquired infections. The increasing prevalence of carbapenem resistant *A. baumannii* has left clinicians with limited treatment options. Last line antimicrobials (i.e., polymyxins and glycylcyclines) are often used as treatment options. The aim of this study was to determine the prevalence of selected β-lactamase genes from *A. baumannii* isolates obtained from patients with hospital-acquired infections and to determine the genetic relationship and epidemiological profiles among clinical *A. baumannii* isolates collected from two tertiary academic hospitals in the Tshwane region, South Africa (SA). Multiplex-PCR (M-PCR) assays were performed to detect selected resistance genes. The collected isolates’ genetic relatedness was determined by using pulsed field gel electrophoresis (PFGE) and multilocus sequence typing (MLST). The acquired oxacillinase (OXA) genes, notably *bla*_OXA-23-like_ were prevalent in the *A. baumannii* isolates. The M-PCR assays showed that the isolates collected from hospital A contained the OXA-23-like (96%; *n* = 69/72) genes and the isolates collected from hospital B contained the OXA-23-like (91%; *n* = 63/69) and OXA-58-like (4%; *n* = 3/69) genes. Colistin resistance was found in 1% of the isolates (*n* = 2/141) and tigecycline intermediate resistance was found in 6% of the isolates (*n* = 8/141). The *A. baumannii* isolates were genetically diverse. Molecular epidemiological data showed that specific sequence types (STs) (ST106, ST229, ST258 and ST208) were established in both hospitals, while ST848 was established in hospital A and ST502, ST339 and the novel ST1552 were established in hospital B. ST848 (established in hospital A) was predominately detected in ICU wards whereas ST208, ST339 and the novel ST1552 (established in hospital B) were detected in ICUs and the general wards. The origin of the *A. baumannii* isolates in the hospitals may be due to the dissemination and adaptation of a diverse group of successful clones. Poor infection control and prevention strategies and possibly the overuse of antimicrobials contributed to the establishment of these *A. baumannii* clones in the studied hospitals.

## Introduction

The incidence of *Acinetobacter baumannii* infections increased over the last decade and unfortunately, so has this bacterium’s antimicrobial resistance (Jacobs et al., 2014). The main mechanisms of β-lactam resistance in *A. baumannii* are the production of carbapenemases, especially Ambler class D β-lactamases and to a lesser extent class B β-lactamases [metallo-β-lactamases (MBLs)] (Kuo et al., 2013; Salimizand et al., 2015; Ghaith et al., 2017). Most of the class D β-lactamases are not inhibited by β-lactamase inhibitors, but are inhibited *in vitro* by sodium chloride (NaCl) (Patel and Bonomo, 2013). The class D β-lactamases comprise of the intrinsic oxacillinase (OXA)-51-like as well as the acquired OXA-23- (OXA-27 and OXA-49); OXA-40- (OXA-25, OXA-26 and OXA-72); OXA-58- (OXA-96 and OXA-97); OXA-143- and OXA-235- (OXA-236 and OXA-237) like enzymes (Kuo et al., 2013; Potron et al., 2015; Ghaith et al., 2017). Insertion sequence (IS) elements play an important role in the mobilisation and expression of OXA-type β-lactamases and in the acquisition of resistance by *A. baumannii* (Evans and Amyes, 2014; Correa et al., 2017). Increased carbapenem resistance in isolates is often linked to IS*AbaI*, as the IS element provides an additional promoter that leads to the overexpression of determinants (Dijkshoorn et al., 2007; Peleg et al., 2008; Evans and Amyes, 2014).

Carbapenemase producing and other multidrug resistant (MDR) *A. baumannii* are extremely difficult to treat and have become a serious problem in healthcare settings due to the limited effective antimicrobial agents available and the association of these resistant strains with poor prognosis (Kuo et al., 2013; Lean et al., 2015). Colistin is often the last line of defence and is used either alone or in combination with other antimicrobials, such as tigecycline and meropenem (Cai et al., 2012; Sekyere, 2016). Colistin resistance among *A. baumannii* is still rare, but resistance and outbreaks have been reported in Italy, Korea and Spain (Agodi et al., 2014; Lean et al., 2014; Lee et al., 2014; Pournaras et al., 2014). Clinical laboratories have been advised to use the micro-broth dilution (MBD) assay to determine colistin susceptibility, since gradient diffusion and disc diffusion are unreliable for this drug class (EUCAST, 2016).

The establishment of clonal relationships among isolates through suitable molecular methods is fundamental for epidemiological surveillance and outbreak investigations as it permits the deployment of effective infection control measures (Cooper and Feil, 2004; Perez et al., 2007; Peleg et al., 2008; Hamouda et al., 2010; Field et al., 2014). The geographical distribution and evolution of the clonal strains can be determined (Grundmann et al., 1997; Field et al., 2014). Different methods can be used, which amongst others include pulsed field gel electrophoresis (PFGE) and multilocus sequence typing (MLST). PFGE is highly discriminatory as minor mutations in outbreak strains can be detected (Fouad et al., 2013; Hu et al., 2013). As MLST is a sequence based method (sequence comparison of internal fragments of housekeeping genes), the global evolutionary relationships between clones can be determined (Cooper and Feil, 2004; Bartual et al., 2005; Fu et al., 2010; Hamouda et al., 2010; Yang et al., 2013; Hammoudi et al., 2014).

The aim of this study was to determine the prevalence of selected antimicrobial resistance genes from patients with hospital-acquired infections and to determine the genetic relationship and epidemiological profiles among clinical *A. baumannii* isolates collected from two tertiary academic hospitals in the Tshwane region, South Africa.

## Materials and Methods

### Isolate Collection, Study Setting and Ethics Statement

One hundred and fifty consecutive non-repeat clinical *A. baumannii* isolates were collected over an 8-month period (October 2013 to May 2014) at the diagnostic division of the Department of Medical Microbiology [Tshwane Academic Division, National Health Laboratory Service (NHLS)]. The laboratory processes specimens from tertiary academic hospitals as well as district hospitals and various clinics as part of standard care. Clinical *A. baumannii* isolates from two tertiary academic hospitals (75 isolates from each hospital) were selected for this study. Hospital A and B have 1 113 beds and 832 beds respectively. A total of nine isolates were excluded from this study, since the isolates tested negative for the OXA-51-like gene (six isolates) or were recovered from the same patient (three isolates). The results are reported on 72 isolates from hospital A and 69 isolates from hospital B (*n* = 141). All the collected isolates were from patients with hospital-acquired infections (conditions that developed 48 h after hospital admission).

This study received ethical approval from the Research Ethics Committee (REC), Faculty of Health Sciences, University of Pretoria (Protocol No. 71/2014). Informed consent was waivered by the REC, since the study was observational and patient care was not influenced.

### Isolate Identification, Confirmation and Antimicrobial Susceptibility Profiles

The isolates were identified and tested for antimicrobial susceptibility using the VITEK^®^ 2 automated system (bioMérieux, Marcy-l’Étoile, France) with the VITEK^®^ 2 GN card and the VITEK^®^ 2 AST-N255 card. Multidrug resistance was defined as bacteria non-susceptible to one or more antimicrobial agents in three or more antimicrobial categories (Magiorakos et al., 2012). All the isolates were screened for the intrinsic OXA-51-like gene (Feizabadi et al., 2008; Voets et al., 2011; Ghaith et al., 2017). The six excluded isolates that did not harbour the OXA-51-like gene were further analysed using matrix assisted laser desorption ionisation-time of flight mass spectrometry (MALDI-TOF). The VITEK^®^ 2 (bioMérieux, Marcy-l’Étoile, France) results for colistin resistant isolates were confirmed by the clinical microbiology laboratory (TAD, NHLS) using a MBD assay (EUCAST, 2016; Clinical and Laboratory Standards Institute [CLSI], 2017).

### Molecular Detection of Beta-Lactamase Resistance Genes

The genomic DNA of each of the 150 *A. baumannii* isolates was extracted as described elsewhere (Lowings et al., 2015). Multiplex PCR assays were performed for the detection of selected β-lactamase genes in *A. baumannii*. Primer concentrations used in the 10x primer mixture and the cycling conditions are shown in Supplementary Table S1.

Primers for IS*AbaI* as previously described by Turton et al. (2006) were used to detect the presence of IS*AbaI*, whereas the IS*AbaI* forward primer and the OXA-23-like, OXA-51-like and OXA-58-like reverse primers (Supplementary Table S1) were used to confirm the presence of IS*AbaI* upstream of the carbapenemase gene (Turton et al., 2006; Voets et al., 2011; Cicek et al., 2014).

### Molecular Epidemiology of the Clinical *A. baumannii* Isolates

The PulseNet PFGE protocol (Ribot et al., 2006) was followed with some modifications as described elsewhere (Lowings et al., 2015). A distance matrix was constructed using the Dice coefficient and a dendrogram was constructed using the unweighted pair group method with arithmetic mean (UPGMA) (Fillaux et al., 2006). Pulsotype designation was based on isolates showing ≥80% relatedness, which corresponds to the Tenover criteria (possibly related 4 to 6 bands difference) (Tenover et al., 1995). Representatives from each major PFGE pulsotype (≥5 isolates) and selective minor pulsotypes (<5 isolates) with ≥80% similarity were chosen for MLST analyses.

Seven housekeeping genes were used for MLST as previously described^1^. All the amplicons were sequenced in both forward and reverse directions by Inqaba Biotechnical Industries, Pretoria, South Africa. The ABI files obtained were analysed using the CLC Main Workbench Version 6.0 (CLCbio, Waltham, MA, United States) software programme. The sequences were assigned to the corresponding allelic profiles and sequence types by using the pubMLST database.

### Statistical Analysis

Analysis was performed with the STATA 14 package (StataCorp LP, College Station, TX, United States). The results were interpreted as follows: *p*-values of ≤ 0.05 were considered statistically significant and *p*-values of >0.1 were considered not significant.

## Results

### Isolate Information

The mean age of patients in hospital A and B were 40 and 41 years, respectively. There was no significant difference between male (M) and female (F) patients in both hospitals [*p*-value = 1.0; M (hospital A): 54% (*n* = 39/72) vs. M (hospital B): 54% (*n* = 37/69)]. More detail on the patient demographics and specimen collection sites can be found in Supplementary Tables S2.1, S2.2.

### Susceptibility Profiles of *A. baumannii* Isolates Isolated From Two Tertiary Academic Hospitals

The susceptibility profiles of the *A. baumannii* isolates collected from both hospitals were determined with the VITEK^®^ 2 automated system (bioMérieux, Marcy-l’Étoile, France) and are represented in Table 1. All the A. *baumannii* isolates were MDR and showed complete resistance toward nitrofurantoin. High levels of resistance toward ceftazidime (90%; *n* = 127/141), cefepime (81%; *n* = 114/141), imipenem (89%; *n* = 125/141), meropenem (87%; *n* = 123/141), gentamicin (74%; *n* = 105/141), ciprofloxacin (67%; *n* = 95/141) and trimethoprim/sulfamethoxazole (69%; *n* = 129/141) were also observed. The majority of the A. baumannii isolates collected from both hospitals were susceptible to tigecycline. However, seven isolates (10%; *n* = 7/72) from hospital A and one isolate (1%; *n* = 1/69) from hospital B showed intermediate resistance toward tigecycline. Two colistin resistant isolates (3%; *n* = 2/69) were isolated from two different patients in hospital B. The results were confirmed by MBD (patient 1: 16 μg mL^-1^ and patient 2: 64 μg mL^-1^).

**Table 1:** Antimicrobial susceptibility profiles of *A. baumannii* isolates collected from hospital A and B.

Number of isolates	Ampicillin	Amoxicillin/ Clavulanic acid	Cefuroxime	Cefuroximine Axetil	Cefoxitin	Cefotaxime	Ceftazidime	Cefepime	Imipenem	Meropenem	Amikacin	Gentamicin	Ciprofloxacin	Tigecycline	Nitrofurantoin	Colistin	Trimethoprim/ Sulfamethoxazole	Sequence type
**Hospital A**																		
39	R	R	R	R	R	R	R	R	R	R	S	R	R	S	R	S	R	106
6	R	R	R	R	R	R	R	R	R	R	S	R	S	S	R	S	R	–
6	R	R	R	R	R	R	R	I	R	R	S	R	R	S	R	S	R	229
6	R	R	R	R	R	R	R	R	R	R	S	S	R	S	R	S	R	848
5	R	R	R	R	R	R	R	R	R	R	S	S	R	I	R	S	R	848
3	R	R	R	R	R	R	S	S	S	S	S	S	S	S	R	S	R	–
2	R	R	R	R	R	R	R	R	R	R	S	R	R	I	R	S	R	–
1	R	R	R	R	R	R	R	R	R	R	S	R	I	S	R	S	R	258
1	R	R	R	R	R	R	R	I	R	I	S	R	R	S	R	S	R	229
1	R	R	R	R	R	R	R	R	I	I	S	R	S	S	R	S	R	–
1	R	R	R	R	R	R	S	S	S	I	S	S	S	S	R	S	R	–
1	R	R	R	R	R	R	S	S	S	S	S	S	S	S	R	S	S	–
**Hospital B**																		
18	R	R	R	R	R	R	R	R	R	R	S	R	R	S	R	S	R	502
16	R	R	R	R	R	R	R	R	R	R	S	R	S	S	R	S	R	339; 1552
8	R	R	R	R	R	R	R	R	R	R	S	I	R	S	R	S	R	208
8	R	R	R	R	R	R	S	S	S	S	S	S	S	S	R	S	S	–
5	R	R	R	R	R	R	R	R	R	R	S	R	I	S	R	S	R	–
4	R	R	R	R	R	R	R	I	R	R	S	R	R	S	R	S	R	–
3	R	R	R	R	R	R	R	R	R	R	I	R	R	S	R	S	R	–
2	R	R	R	R	R	R	R	R	R	R	S	S	S	S	R	S	R	–
1	R	R	R	R	R	R	R	R	R	R	S	S	S	S	R	R	R	–
1	R	R	R	R	R	R	R	R	R	R	S	R	R	I	R	S	R	–
1	R	R	R	R	R	R	R	I	R	I	S	R	R	S	R	S	S	–
1	R	R	R	R	R	R	S	S	S	S	S	R	S	S	R	R	R	–
1	R	R	R	R	R	R	R	R	R	R	R	S	R	S	R	S	R	–

*R, resistant; I, intermediate resistant; S, susceptible; –, no data.*

### Molecular Identification of Resistance Genes Found in *A. baumannii* Isolates Using M-PCR Assays

All the collected isolates were screened for the intrinsic OXA-51-like gene (Feizabadi et al., 2008; Voets et al., 2011; Ghaith et al., 2017). Six isolates were excluded that did not contain this intrinsic gene. The rest of the isolates all contained the OXA-51-like gene (hospital A: 100%; *n* = 72/72 and hospital B: 100%; *n* = 69/69). The isolates collected from hospital A contained the OXA-23-like (96%; *n* = 69/72) gene and the isolates collected from hospital B contained the OXA-23-like (91%; *n* = 63/69) and OXA-58-like (4%; *n* = 3/69) genes.

The 141 *A. baumannii* isolates were screened for IS*AbaI* and 100% (*n* = 72/72) of the isolates from hospital A and 88% (*n* = 61/69) of the isolates in hospital B contained this element. The detection of the IS*AbaI* was statistically significant [*p-*value = 0.003]. Further tests were performed to determine if the element was upstream of the OXA genes. Only 29% (*n* = 21/72) of the isolates in hospital A and 42% (*n* = 29/69) of the isolates in hospital B carried the IS*AbaI* upstream of the OXA-51-like gene. These findings were not statistically significant [*p-*value = 0.118] and no increased MICs for carbapenems were observed. The IS*AbaI* element was not detected upstream of either the OXA-23-like or the OXA-58-like genes in any of the isolates.

The Temoneira (TEM) gene was detected in 33% (*n* = 24/72) and 17% (*n* = 12/69) of the isolates collected from hospital A and B respectively. There was a significant association of the TEM gene between the hospitals [*p-*value = 0.035]. The *A. baumannii* isolates were negative for the following genes: Cefotaximase-Munich (CTX-M); Guiana extended-spectrum β-lactamase (GES); German imipenemase (GIM); Imipenem metallo-β-lactamase (IMP); *Klebsiella pneumoniae* carbapenemase (KPC); New Delhi metallo-β-lactamase (NDM), OXA-48; *Pseudomonas* extended resistance (PER); Sulfhydryl variant (SHV), Seoul imipenem metallo-β-lactamase (SIM-1); São Paulo metallo-β-lactamase (SPM), Vietnam extended-spectrum β-lactamase (VEB) and Verona integrin-encoded metallo-β-lactamase (VIM). The mechanisms of colistin and tigecycline resistance were not further investigated.

### Determination of the Genetic Relationship and Global Epidemiology of Clinical *A. baumannii* Isolates

The PFGE analysis clustered the *A. baumannii* isolates into seven major pulsotypes (≥5 isolates) and several minor pulsotypes (<5 isolates) (Supplementary Figure S1). Twenty representative *A. baumannii* isolates were chosen from each major pulsotype (top and bottom isolates from each pulsotype) and selected minor pulsotypes. The following STs were identified according to the Oxford database: ST106 (1-1-1-1-1-98-6); ST208 (1-3-3-2-2-97-3); ST229 (1-15-2-28-1-107-32); ST258 (1-15-8-10-28-110-32); ST339 (44-73-4-11-44-121-4); ST502 (1-12-3-2-2-100-3); ST848 (1-15-3-2-2-142-3); and ST1552 (44-73-4-11-44-304-4). Molecular epidemiological data revealed that ST106, ST229, ST258 and ST208 are established in both hospitals. ST848 was only detected in hospital A, while ST502, ST339 and the novel ST1552 were only detected in hospital B.

## Discussion

**FIGURE 1:**
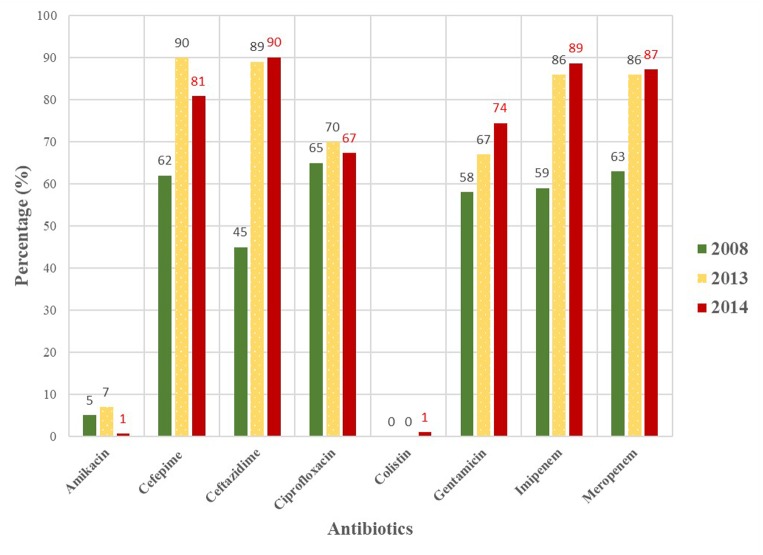
Antimicrobial susceptibility profiles of MDR *Acinetobacter baumannii* isolates collected from hospitals in the Tshwane region in 2008, 2013 and 2014 (Kock et al., 2013; Lowings et al., 2015).

The MDR *A. baumannii* isolates pose great treatment challenges in the studied hospitals. When the data from this study is compared to that of previously published studies (Kock et al., 2013; Lowings et al., 2015) in the same hospitals, the data suggests that the problem is only set to increase. In **Figure 1** the steady increase in antimicrobial resistance since 2008 in Tshwane, South Africa is depicted. The proportion of resistance increased alarmingly from 2008 to 2013; not much changed as similar resistance profiles were observed in 2014 (this study). The detection of colistin resistance and intermediate tigecycline resistance is worrisome as these are last resort antimicrobials. Resistance toward these antimicrobials are likely to increase in the years to come if it is not used as prescribed by local and international guidelines (Cai et al., 2012; Sekyere, 2016).

Circulating antimicrobial resistance genes can be determined with molecular assays, such as PCR, which are relatively fast and cheap. The PCR results showed that the majority of the collected isolates were positive for the OXA-51-like gene except for six isolates that tested negative (these isolates were excluded from the study). These six isolates were identified by MALDI-TOF as: (i) *Acinetobacter junii*; (ii) *A. pittii* (x2); (iii) *A. haemolyticus*; (iv) *A. nosocomialis* and (v) *Providencia rettgeri*. The PCR and MALDI-TOF results have shown that the presence of the intrinsic/species-specific OXA-51-like gene can be used as a simple and reliable way to identify *A. baumannii* (Turton et al., 2006; Feizabadi et al., 2008; Voets et al., 2011; Ghaith et al., 2017). The prevalence of the OXA-23-like gene was very high in this study and have increased with 35% since 2008 to 2014 (Kock et al., 2013; Lowings et al., 2015). The drastic increase in the OXA-23 gene could be due to poor infection control practises in the studied hospitals. Isolates that harboured this gene showed higher resistance levels toward carbapenems, such as imipenem and meropenem. The high prevalence of the OXA-23 gene in this study is in agreement with other studies done in India (98%; *n* = 101/103), Colombia (97.5%; *n* = 118/121) and Egypt (96%; *n* = 48/50) (Vijayakumar et al., 2016; Correa et al., 2017; Ghaith et al., 2017). The prevalence of the OXA-58-like gene remains low in the studied hospitals and no drastic increase were observed over the years (Kock et al., 2013; Lowings et al., 2015). However, Mendes et al. (2009) and Liakopoulos et al. (2012) reported higher OXA-58 carriage rates [12% (Asia-Pacific nations) and 27.6% (Greece) respectively]. The TEM gene, classified as an extended spectrum β-lactamase (ESBL) from the Ambler class A group, was detected in some of the collected isolates. The TEM gene was the most frequently associated with ST848, followed by ST106 and ST208. The detection of the TEM gene in the studied hospitals shows how the *A. baumannii* clones are constantly evolving by gaining more genetic determinants from other Gram-negative bacteria.

Molecular typing methods, such as PFGE and MLST are useful in epidemiological studies to identify circulating clones (Villalón et al., 2011). A literature research was done on the eight clones detected in this study and the distributions of these clones are summarised in **Table 2**. Sequence type 106 was the most frequently detected clone followed by ST258 (both of these clones were only susceptible to amikacin, colistin and tigecycline). It appears that the ST258 clone is a South African clone (Lowings et al., 2015) as no other reports could be found. This is to our knowledge the first report of the presence of ST1552 – a single locus variant of ST339. This highlights the constant evolution and dissemination of *A. baumannii* clones in and between hospital settings. The global clone ST208 was also detected in this study (Adams-Haduch et al., 2011). These successful clones have increased virulence and survival factors and are adapted to survive in the harsh hospital environment. These circulating clones poses an increased threat to hospitalised patients and stricter adherence to infection control and prevention policies are needed to prevent further dissemination of these MDR clones. The general cleaning of the hospital environment should also be improved as it is possible for these clones to survive on abiotic surfaces, such as medical equipment, furniture, door handles and bed linen (Maragakis and Perl, 2008).

**Table 2:** The global epidemiology of the eight detected STs in this study.

ST	Number of isolates detected in this study (*n* = 20)	Hospital epidemiology	Reported countries	Reference
106	6	General ICUs, surgery ICUs and high care units	Greece, Japan and South Africa	Endo et al., 2012; Liakopoulos et al., 2012; Lowings et al., 2015; Mavroidi et al., 2015
848	4	ICU	Iran and South Africa	Lowings et al., 2015; Saffari et al., 2017
208	2	Medical and pulmonary ICU, radiation oncology ward, high care multidisciplinary unit and internal medicine female ward	Asia, Europe and the United States as well as in Mexico, Saudi Arabia and South Africa^∗^	Adams-Haduch et al., 2011; Tan et al., 2013; Asai et al., 2014; Lee et al., 2015; Sousa et al., 2015; Zowawi et al., 2015; Fang et al., 2016; Qu et al., 2016; Tamayo-Legorreta et al., 2016; Kim et al., 2017; Pfeifer et al., 2017; Jeon et al., 2018; This study
229	2	Predominantly detected in ICU wards with two isolates being detected in surgery and trauma wards	China, Egypt, Saudi Arabia and South Korea	Wu et al., 2015; Zowawi et al., 2015; Huang et al., 2016; Ghaith et al., 2017; Kim et al., 2017; Jeon et al., 2018
502	2	Surgery ICU	Bulgaria and South Africa	Lowings et al., 2015; Pfeifer et al., 2017
1552	2	Pulmonology ICU	South Africa^∗^	This study
258	1	Paediatric surgery wards, neonatal ICU and surgery ICU	South Africa	Lowings et al., 2015
339	1	Medical and pulmonary ICUs, orthopaedic elective ward, high care units and internal medicine female ward	Brazil and South Africa	Clímaco et al., 2013; Lowings et al., 2015

*^∗^Detected in this study.*

Limitations of this study include: (i) small sample size; (ii) only two tertiary hospitals in the Tshwane region were included and (iii) there is a lack of detailed clinical information, such as treatment received, clinical outcomes and other simultaneous co-morbidities and infections. The investigation of the different antimicrobial resistance mechanisms was not part of the scope of this study. Future studies, employing whole genome sequencing and other next generation technologies, should investigate the antimicrobial resistance mechanisms linked to the colistin and tigecycline resistance observed.

## Conclusion

The high prevalence of MDR *A. baumannii* isolates is threatening the effectiveness of last resort antimicrobials. The detection of colistin resistance and intermediate tigecycline resistance is of concern, since no other treatment options are available if these drugs are rendered ineffective. A diverse group of highly resistant clones with the OXA-23-like gene is responsible for the spread and establishment of *A. baumannii* in the studied hospitals. The spread of *A. baumannii* clones between the studied hospitals could be due to patients being transferred from one hospital to the other (the hospitals are in close proximity) or it could be due to healthcare workers rotating between hospital A and B (both hospitals are part of the same academic institution). Future studies incorporating next generation sequencing methods focusing on a larger sample of *A. baumannii* isolates from various hospitals and lineages are necessary to better understand the rapid development of antimicrobial resistance in *A. baumannii*.

## Author Contributions

ML, ME, and MK conceived and designed the study. MK was the principal investigator and budget owner. ML collected all the clinical isolates and performed the experimental work. ML, GP, and MK performed the data analysis. ML and FI collected all the clinical information. ML, ME, FI, GP, PB, JP, and MK wrote the manuscript with critical appraisal and contributions received from all of the authors. PB did the statistical analysis. All authors read and approved this version of the manuscript.

## Conflict of Interest Statement

The authors declare that the research was conducted in the absence of any commercial or financial relationships that could be construed as a potential conflict of interest. The reviewer VG and handling Editor declared their shared affiliation.

## Footnotes

1 https://pubmlst.org/abaumannii/info/primers_Oxford.shtml
