# Supplementary material for: Acinetobacter baumannii: Epidemiological and Beta-Lactamase Data From Two Tertiary Academic Hospitals in Tshwane, South Africa
Source: Front Microbiol. 2018 Jun 12;9:1280. doi: 10.3389/fmicb.2018.01280 (PMC6005857; doi:10.3389/fmicb.2018.01280)
Supplement: Supplementary file 1 [file Table_1.pdf]

Supplementary Material

Acinetobacter baumannii: epidemiological and β-lactamase data from two tertiary academic hospitals in Tshwane, South Africa

M. Lowe, M.M. Ehlers, F. Ismail, G. Peirano, P.J. Becker, J.D.D. Pitout, M.M. Kock*

*Correspondence: marleen.kock@up.ac.za

Table S1: Primer nucleotide sequences of antimicrobial resistance genes in clinical *A. baumannii* isolates

Target	Primer name	Primer sequence (5' to 3')#	Amplicon size (bp)	Primer concentration used (µM)	Reference
**Multiplex I**:					
CTX-M	CTX-M-U (F)	ATGTGCAGYACCAGTAARGTKATGGC	593	0.25	Monstein *et al*., 2007
	CTX-M-U (R)	TGGGTRAARTARGTSACCAGAAYCAGCGG			
SHV	bla_{SHV} (F)	AGCCGCTTGAGCAAATTAAAC	713	0.2	Dallenne *et al*., 2010
	bla_{SHV} (R)	ATCCCGCAGATAAATCACCAC			
TEM	TEM (F)	TGCCGCATACACTATTCTCAGAATGA	445	0.3	Monstein *et al*., 2007
	TEM (R)	ACGCTCACCGGCCTCCAGATTTAT			
**Multiplex II**:					
IMP	bla_{IMP} (F)	TTGACACTCCATTTACDG**	139	0.2	Dallenne *et al*., 2010
	bla_{IMP} (R)	GATYGAGAATTAAGCCACYCT			
KPC	bla_{KPC} (F)	CATTCAAGGGCTTTCTTGCTGC	538	0.2	
	bla_{KPC} (R)	ACGACGGCATAGTCATTTGC			
VIM	bla_{VIM} (F)	GATGGTGTTTGGTCGCATA	390	0.5	
	bla_{VIM} (R)	CGAATGCGCAGCACCAG			
**Multiplex III**:					
GES	bla_{GES} (F)	AGTCGGCTAGACCGGAAAG	399	0.3	Dallenne *et al*., 2010
	bla_{GES} (R)	TTTGTCCGTGCTCAGGAT			
PER	bla_{PER} (F)	GCTCCGATAATGAAAGCGT	520	0.3	
	bla_{PER} (R)	TTCGGCTTGACTCGGCTGA			
VEB	bla_{VEB} (F)	CATTTCCCGATGCAAAGCGT	648	0.3	
	bla_{VEB} (R)	CGAAGTTTCTTTGGACTCTG			
**Multiplex IV**:					
GIM	bla_{GIM} (F)	CGTTGCCAGCTTTAGCTCAGG	279	0.4	Voets *et al.*, 2011
	bla_{GIM} (R)	GCAACTTGATACCAGCAGTGCG			
NDM-1	$bla_{\mathrm{NDM\text{-}1}}$(F)	CCCGGCCACACCAGTGACA	129	0.7	
	$bla_{\mathrm{NDM\text{-}1}}$(R)	GTAGTGCTCAGTGTCGGCAT			
SIM-1	bla_{SIM} (F)	TTGCGGAAGAAGCCCAGCCAG	613	0.4	
	bla_{SIM} (R)	GCGTCTCCGATTTCACTGTGGC			
SPM	bla_{SPM} (F)	GGGTGGCTAAGACTATGAAGCC	447	1.3	
	bla_{SPM} (R)	GCCGCCGAGCTGAATCGG			
**Multiplex V**:					
OXA-23-like	$bla_{\mathrm{OXA\text{-}23}}$ (F)	GATCGGATTGGAGAACCAGA	501	0.2	Woodford *et al*., 2006
	$bla_{\mathrm{OXA\text{-}23}}$ (R)	ATTTCTGACCGCATTTCCAT			
OXA-48	$bla_{\mathrm{OXA\text{-}48}}$ (F)	GCGTGGTTAAGGATGAACAC	438	0.2	
	$bla_{\mathrm{OXA\text{-}48}}$ (R)	CATCAAGTTCAACCCAACCG			
OXA-51-like	$bla_{\mathrm{OXA\text{-}51}}$ (F)	TAATGCTTTGATCGGCCTTG	353	0.2	
	$bla_{\mathrm{OXA\text{-}51}}$ (R)	TGGATTGCACTTCATCTTGG			
OXA-58-like	$bla_{\mathrm{OXA\text{-}58}}$ (F)	AAGTATTGGGGCTTGTGCTG	599	0.2	
	$bla_{\mathrm{OXA\text{-}58}}$ (R)	CCCCTCTGCGCTCTACATAC			

**Y=T or C; D=A or G or T; # All oligonucleotides were synthesised and purified by Inqaba Biotechnical Industries, Pretoria, South Africa

Positive controls:

The positive controls used included three ATCC cultures, namely *A. baumannii* ATCC BAA-1605 (*bla*$_{OXA-23}$ and *bla*$_{OXA-51}$); *K. pneumoniae* ATCC 8303 [*Klebsiella pneumoniae* carbapenemase (KPC) positive control] and *K. pneumoniae* ATCC BAA-2146 [New Delhi metallo-β-lactamase (NDM) positive control] as well as local clinical isolates that had been identified and sequenced in former departmental studies. The local clinical isolates used included positive controls for the following genes: Cefotaximase-Munich (CTX-M); Guyana extended-spectrum β-lactamase (GES); German imipenemase (GIM); Imipenem metallo-β-lactamase (IMP); *Pseudomonas* extended resistance (PER); Sulfhydryl variant (SHV); Seoul imipenem metallo-β-lactamase (SIM); Sao Paulo metallo-β-lactamase (SPM); Temoneira (TEM); Vietnam extended-spectrum β-lactamase (VEB) and Verona integrin-encoded metallo-β-lactamase (VIM).

Cycling conditions for multiplex I to V:

An initial denaturation step of 95°C for 5 min, followed by 30 cycles of denaturation at 95°C for 30 sec, an annealing temperature dependent on the melting temperature of the primer pair (Multiplex I and IV 60°C; Multiplex II 55 °C; Multiplex III and V 56°C) and extension at 72°C for 90 sec, followed by the final extension step at 70°C for 10 min. Positive and negative controls were included in all the M-PCR assays.

Cycling conditions for IS*Aba*I):

The cycling conditions were followed as described by Segal *et al.* (2005) with one modification; an annealing temperature of 52°C was used.

References:

Dallenne, C., Da Costa, A., Decré, D., Favier, C., Arlet, G. (2010). Development of a set of multiplex PCR assays for the detection of genes encoding important β-lactamases in *Enterobacteriaceae*. *J Antimicrob Chemother* 65, 490-495

Monstein, H.J., Östholm-Balkhed, Å., Nilsson, M.V., Nilsson, M., Dornbusch, K., Nilsson, L.E. (2007). Multiplex PCR amplification assay for the detection of *bla*$_{SHV}$, *bla*$_{TEM}$ and *bla*$_{CTX-M}$ genes in *Enterobacteriaceae*. *APMIS* 12, 1400-1408

Segal, H., Garny, S., Elisha, B.G. (2005). Is IS*Aba-1* customized for *Acinetobacter*? *FEMS Microbiol Lett* 243, 425-429

Voets, G.M., Fluit, A.C., Scharringa, J., Stuart, J.C., Leverstein-Van Hall, M.A. (2011). A set of multiplex PCRs for genotypic detection of extended-spectrum β-lactamases, carbapenemases, plasmid-mediated AmpC β-lactamases and OXA β-lactamases. *Int J Antimicrob Agents* 37, 356-359

Woodford, N., Ellington, M.J., Coelho, J.M., Turton, J.F., Ward, M.E., Brown, S., et al. (2006). Multiplex PCR for genes encoding prevalent OXA carbapenemases in *Acinetobacter* spp. *Int J Antimicrob Agents* 27, 351-353
