# Supplementary material for: Acinetobacter baumannii: Epidemiological and Beta-Lactamase Data From Two Tertiary Academic Hospitals in Tshwane, South Africa
Source: Front Microbiol. 2018 Jun 12;9:1280. doi: 10.3389/fmicb.2018.01280 (PMC6005857; doi:10.3389/fmicb.2018.01280)
Supplement: Supplementary file 2 [file Table_2.PDF]

Supplementary Material

Acinetobacter baumannii: epidemiological and β-lactamase data from two tertiary academic hospitals in Tshwane, South Africa

M. Lowe, M.M. Ehlers, F. Ismail, G. Peirano, P.J. Becker, J.D.D. Pitout, M.M. Kock*

* **Correspondence**: Prof Marleen Kock: marleen.kock@up.ac.za

Table S2.1: Patient demographics and specimen collection sites (Hospital A)

Hospital	Patient	Ward	Age	Gender	Collected from	ST
**A**	**1**	ICU	65 y	M	Sputum	-
**A**	**2**	Surgery OPD	40 y	F	Fluid/Aspirate	-
**A**	**3**	ICU	44 y	M	Tracheal aspirate	106
**A**	**4**	ICU	60 y	M	Tracheal aspirate	-
**A**	**5**	High Care Unit	23 y	M	Sputum	106
**A**	**6**	ICU	56 y	M	Tracheal aspirate	-
**A**	**7**	Orthopaedics	32 y	F	Swab (superficial)	-
**A**	**8**	Obstetrics & Gynaecology	25 y	F	Swab (superficial)	-
**A**	**9**	Obstetrics & Gynaecology	24 y	F	Clotted blood	106
**A**	**10**	ICU	23 y	F	Blood culture	106
**A**	**11**	High Care Unit	58 y	M	Sputum	848
**A**	**12**	Internal medicine	25 y	M	Fluid/Aspirate	258
**A**	**13**	ICU	60 y	M	Tracheal aspirate	106
**A**	**14**	ICU	37 y	M	Blood culture	229
**A**	**15**	Casualty	42 y	F	Stool	-
**A**	**16**	ICU	23 y	M	Tracheal aspirate	-
**A**	**17**	ICU	33 y	M	Intravenous catheter tip	-
**A**	**18**	ICU	23 y	F	Blood culture	106
**A**	**19**	Medical OPD	68 y	F	Swab (superficial)	106
**A**	**20**	General surgery	54 y	M	Blood culture	229
**A**	**21**	ICU	23 y	F	Blood culture	106
**A**	**22**	ICU	31 y	M	Sputum	106
**A**	**23**	ICU	35 y	F	Intravenous catheter tip	-
**A**	**24**	ICU	35 y	F	Intravenous catheter tip	-
**A**	**25**	Internal medicine	46 y	F	Intravenous catheter tip	848
**A**	**26**	ICU	62 y	M	Intravenous catheter tip	848
**A**	**27**	Gynaecology	25 y	F	Fluid/Aspirate	-
**A**	**28**	ICU	42 y	M	Intravenous catheter tip	848
**A**	**29**	Neonatal	1 y	F	Blood culture	258
**A**	**30**	ICU	62 y	M	Tracheal aspirate	-
**A**	**31**	ICU	62 y	M	Arterial catheter tip	848
**A**	**32**	Gynaecology	25 y	F	Swab (superficial)	-
**A**	**33**	High Care Unit	46 y	F	Bronchial aspirate	-
**A**	**34**	Internal medicine	29 y	F	Intravenous catheter tip	258
**A**	**35**	ICU	19 y	M	Intravenous catheter tip	848
**A**	**36**	ICU	-	F	Tracheal aspirate	-
**A**	**37**	*Excluded*	-	-	-	-

M = Male; F = Female; ICU = Intensive care unit; OPD = Outpatient department; M = Male; F = Female; ICU = Intensive care unit; OPD = Outpatient department; Blue shading = Sequenced isolates; Grey shading = Pulsotype groups with ≥80% similarity

Table S2.1: Patient demographics and specimen collection sites (Hospital A) - continued

Hospital	Patient	Ward	Age	Gender	Collected from	ST
**A**	**38**	*Excluded*	-	-	-	-
**A**	**39**	High Care Unit	33 y	M	Intravenous catheter tip	-
**A**	**40**	Orthopaedics	70 y	F	Swab (superficial)	-
**A**	**41**	Obstetrics & Gynaecology	32 y	F	Swab (superficial)	1552
**A**	**42**	ICU	37 y	F	Urine	848
**A**	**43**	Combined surgical disciplines	-	M	Swab (superficial)	-
**A**	**44**	ICU	46 y	F	Sputum	229
**A**	**45**	ICU	27 y	M	Tracheal aspirate	-
**A**	**46**	Orthopaedics	76 y	M	Urine	258
**A**	**47**	*Excluded*	-	-	-	-
**A**	**48**	ICU	27 y	M	Tracheal aspirate	106
**A**	**49**	ICU	19 y	M	Tracheal aspirate	-
**A**	**50**	Obstetrics & Gynaecology	30 y	F	Swab (superficial)	-
**A**	**51**	Neonatal (High Care)	3 d	F	Blood culture	-
**A**	**52**	High Care Unit	64 y	M	Tracheal aspirate	848
**A**	**53**	ICU	64 y	M	Wound swab	106
**A**	**54**	ICU	35 y	F	Tracheal aspirate	-
**A**	**55**	ICU	19 y	M	Blood culture	106
**A**	**56**	ICU	68 y	M	Tracheal aspirate	-
**A**	**57**	High Care Unit	29 y	M	Tracheal aspirate	-
**A**	**58**	Operating theatre	19 y	M	Tissue	106
**A**	**59**	High Care Unit	75 y	M	Tracheal aspirate	229
**A**	**60**	ICU	45 y	M	Tracheal aspirate	106
**A**	**61**	ICU	30 y	M	Tracheal aspirate	-
**A**	**62**	Internal medicine	35 y	F	Blood culture	-
**A**	**63**	ICU	22 y	F	Intravenous catheter tip	-
**A**	**64**	ICU	35 y	F	Arterial catheter tip	-
**A**	**65**	ICU	35 y	F	Blood culture	106
**A**	**66**	ICU	68 y	M	Tracheal aspirate	106
**A**	**67**	High Care Unit	59 y	M	Tracheal aspirate	-
**A**	**68**	Gynaecology	40 y	F	Urine	106
**A**	**69**	ICU	35 y	F	Intravenous catheter tip	-
**A**	**70**	ICU	37 y	F	Intravenous catheter tip	106
**A**	**71**	High Care Unit	13 y	M	Sputum	106
**A**	**72**	ICU	58 y	M	Tracheal aspirate	-
**A**	**73**	ICU	50 y	M	Tracheal aspirate	-
**A**	**74**	ICU	22 y	F	Blood culture	-
**A**	**75**	General surgery	53 y	M	Blood culture	-

M = Male; F = Female; ICU = Intensive care unit; OPD = Outpatient department; M = Male; F = Female; ICU = Intensive care unit; OPD = Outpatient department; Blue shading = Sequenced isolates; Grey shading = Pulsotype groups with $\geq$80% similarity

Table S2.2: Patient demographics and specimen collection sites (Hospital B)

Hospital	Patient	Ward	Age	Gender	Collected from	ST
B	1	Medical & Pulmonology ICU	18 y	M	Tracheal Aspirate	1552
B	2	Paediatric Surgery Ward	11 y	M	Arterial catheter tip	-
B	3	Medical & Pulmonology ICU	20 y	F	Tracheal Aspirate	208
B	4	Surgery ICU	78 y	F	Tracheal Aspirate	-
B	5	Medical & Pulmonology ICU	62 y	F	Arterial catheter tip	-
B	6	Medical & Pulmonology ICU	59 y	F	Broncho-alveolar lavage	-
B	7	Medical & Pulmonology ICU	20 y	F	Sputum	-
B	8	Neurosurgical Ward	37 y	M	Tracheal Aspirate	-
B	9	Surgery ICU	76 y	F	Tracheal Aspirate	339
B	10	Coronary ICU	66 y	M	Blood culture	-
B	11	*Excluded*	-	-	-	-
B	12	*Excluded*	-	-	-	-
B	13	Internal Medicine Female Ward	33 y	F	Fluid/Aspirate	339
B	14	Neurosurgery ICU	42 y	M	Sputum	-
B	15	Surgery ICU	32 y	F	Tracheal Aspirate	-
B	16	Oncology Ward	11 y	F	Swab	-
B	17	Surgery ICU	56 y	M	Tracheal Aspirate	1552
B	18	Surgery ICU	62 y	F	Sputum	-
B	19	Neurosurgery ICU	60 y	M	Tracheal Aspirate	-
B	20	Medical & Pulmonology ICU	59 y	F	Intravenous catheter tip	1552
B	21	Medical & Pulmonology ICU	57 y	M	Tracheal Aspirate	1552
B	22	Medical & Pulmonology ICU	37 y	M	Tracheal Aspirate	-
B	23	Coronary ICU	65 y	M	Intravenous catheter tip	-
B	24	Urology & Gynaecology	45 y	F	Clotted blood/PPT tube	-
B	25	Paediatric Surgery Ward	9 d	M	Blood culture	-
B	26	Orthopaedic Elective Ward	28 y	M	Swab	-
B	27	Medical & Pulmonology ICU	31 y	F	Tracheal Aspirate	1552
B	28	Radiation Oncology	34 y	F	Urine	-
B	29	Internal Medicine Female Ward	61 y	F	Urine	208
B	30	Surgery ICU	41 y	M	Blood culture	-
B	31	*Excluded*	-	-	-	-
B	32	Surgery ICU	54 y	F	Tracheal Aspirate	106
B	33	Neurosurgery ICU	30 y	M	Tracheal Aspirate	-
B	34	Surgery ICU	41 y	M	Blood culture	502
B	35	Medical & Pulmonology ICU	37 y	M	Arterial catheter tip	-
B	36	Medical & Pulmonology ICU	55 y	M	Arterial catheter tip	-
B	37	Coronary ICU	62 y	F	Arterial catheter tip	-
B	38	*Excluded*	-	-	-	-
B	39	Orthopaedic Trauma	16 y	M	Tissue	-
B	40	High Care Multidiscipline	27 y	M	Blood culture	-
B	41	Paediatric Medical ICU	2 y	M	Blood culture	-
B	42	High Care Multidiscipline	27 y	M	Blood culture	-
B	43	Surgery ICU	56 y	M	Tracheal Aspirate	-
B	44	Paediatric Surgery Ward	15 d	M	Blood culture	258
B	45	Neurosurgery ICU	65 y	F	Sputum	-
B	46	Neonatal ICU	11 d	F	Blood culture	-
B	47	Internal Medicine Female Ward	34 y	F	Midstream urine	-
B	48	*Excluded*	-	-	-	-

M = Male; F = Female; ICU = Intensive care unit; Blue shading = Sequenced isolates; Grey shading = Pulsotype groups with ≥80% similarity

Table S2.2: Patient demographics and specimen collection sites (Hospital B) - continued

Hospital	Patient	Ward	Age	Gender	Collected from	ST
**B**	**49**	Medical & Pulmonology ICU	45 y	M	Tracheal Aspirate	-
**B**	**50**	*Excluded*	-	-	-	-
**B**	**51**	Orthopaedic Elective Ward	34 y	F	Urine	339
**B**	**52**	Main Casualty	44 y	F	Midstream urine	-
**B**	**53**	Medical & Pulmonology ICU	69 y	F	Broncho-alveolar lavage	-
**B**	**54**	Neurosurgery ICU	28 y	M	Tracheal Aspirate	339
**B**	**55**	Admissions	19 y	F	Fluid/Aspirate	-
**B**	**56**	Medical & Pulmonology ICU	61 y	M	Arterial catheter tip	258
**B**	**57**	High Care Multidiscipline	46 y	F	Blood culture	-
**B**	**58**	Surgery ICU	26 y	M	Tracheal Aspirate	-
**B**	**59**	Surgery ICU	85 y	F	Tracheal Aspirate	-
**B**	**60**	Neurosurgery ICU	59 y	M	Tracheal Aspirate	229
**B**	**61**	Surgery ICU	22 y	M	Tracheal Aspirate	-
**B**	**62**	Surgery ICU	59 y	F	Intravenous catheter tip	-
**B**	**63**	Nephrology/Peritoneal Ward	18 y	M	Intravenous catheter tip	502
**B**	**64**	High Care Multidiscipline	61 y	M	Sputum	-
**B**	**65**	High Care Multidiscipline	31 y	F	Swab	106
**B**	**66**	Surgery ICU	58 y	F	Blood culture	339
**B**	**67**	Paediatric Surgery Ward	1 y	F	Intravenous catheter tip	-
**B**	**68**	Medical & Pulmonology ICU	54 y	M	Sputum	339
**B**	**69**	Paediatric Surgery Ward	1 y	F	Intravenous catheter tip	-
**B**	**70**	Medical & Pulmonology ICU	41 y	M	Tracheal Aspirate	-
**B**	**71**	Neurosurgery ICU	44 y	M	Tracheal Aspirate	229
**B**	**72**	Internal Medicine Female Ward	65 y	F	Swab	-
**B**	**73**	Surgery ICU	56 y	M	Tracheal Aspirate	-
**B**	**74**	Surgery ICU	19 y	M	Sputum	258
**B**	**75**	Internal Medicine Male Ward	57 y	M	Sputum	-

M = Male; F = Female; ICU = Intensive care unit; Blue shading = Sequenced isolates; Blue shading = Sequenced isolates; Grey shading = Pulsotype groups with $\geq$80% similarity
