# Supplementary material for: Acinetobacter baumannii: Epidemiological and Beta-Lactamase Data From Two Tertiary Academic Hospitals in Tshwane, South Africa
Source: Front Microbiol. 2018 Jun 12;9:1280. doi: 10.3389/fmicb.2018.01280 (PMC6005857; doi:10.3389/fmicb.2018.01280)
Supplement: Supplementary file 3 [file Image_1.PDF]

Supplementary Material

Acinetobacter baumannii: epidemiological and β-lactamase data from two tertiary academic hospitals in Tshwane, South Africa

M. Lowe, M.M. Ehlers, F. Ismail, G. Peirano, P.J. Becker, J.D.D. Pitout, M.M. Kock*

*Correspondence: marleen.kock@up.ac.

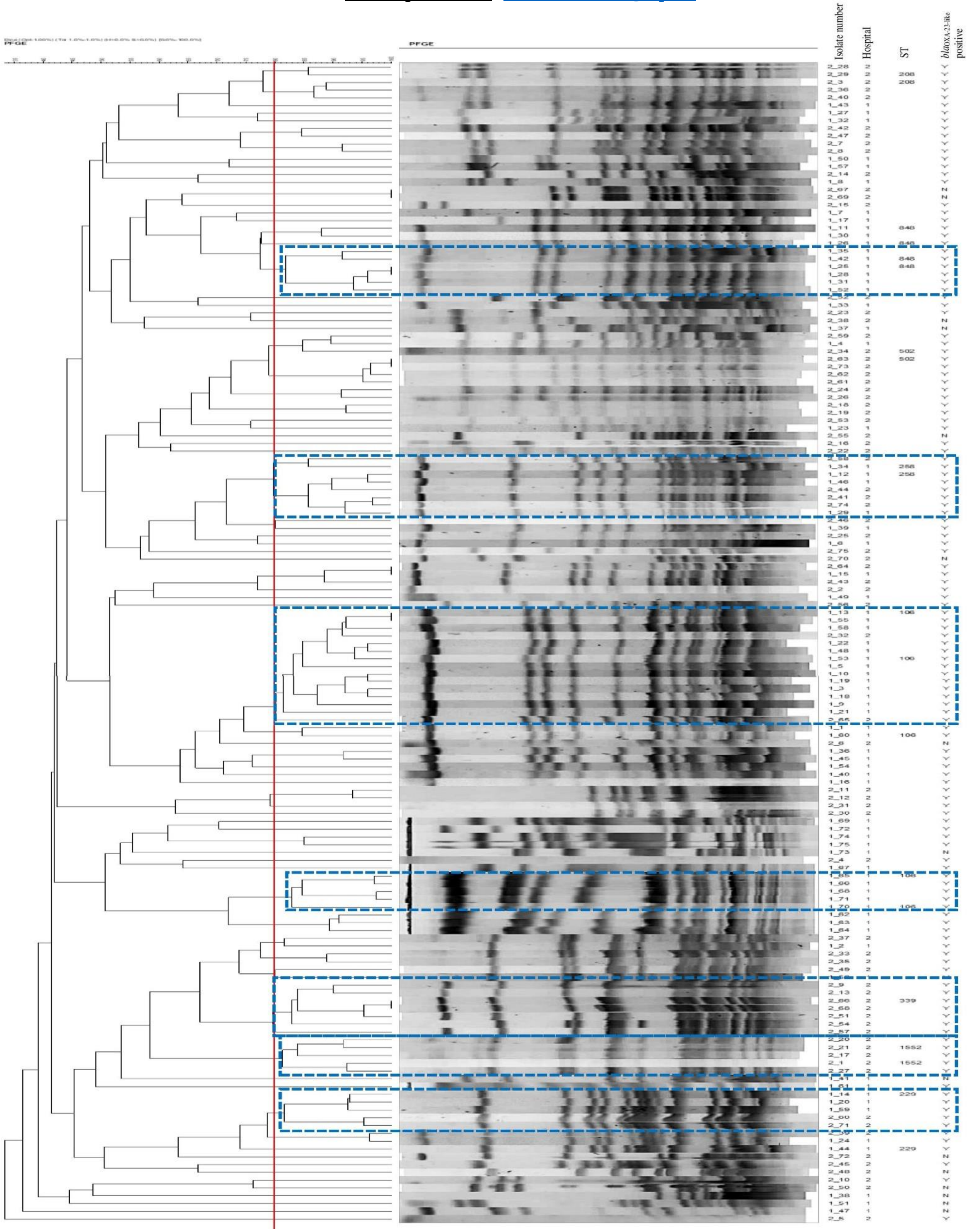

Figure S1: The PFGE patterns of *A. baumannii* isolates

The banding patterns were analysed using GelComparII (Applied Maths, Belgium). A distance matrix was constructed using the Dice coefficient and a dendrogram was constructed from the distance matrix using the unweighted pair group method with arithmetic mean (UPGMA). Major pulsotype designation was based on five or more isolates showing ≥80% relatedness. The seven major pulsotype groups identified are indicated. Isolate name, hospital (1=hospital A; 2=hospital B), ST and the carriage of *bla*$_{OXA-23}$ (Y=positive; N=negative) are indicated.
